# Supplementary material for: A homozygous FANCM mutation underlies a familial case of non-syndromic primary ovarian insufficiency
Source: eLife. 2017 Dec 12;6:e30490. doi: 10.7554/eLife.30490 (PMC5764568; doi:10.7554/eLife.30490)
Supplement: Figure 3—source data 1. — Numbers are the percentage of cell growth in treated samples compared to the untreated cells. [file elife-30490-fig3-data1.doc]

| **EXP. 1** |  |  |  |  |  |  |
| --- | --- | --- | --- | --- | --- | --- |
| Dose MMC | Affected | Mother | FAA |  | FAC | WT |
|  | 0 | 100 | 100 | 100 | 100 | 100 |
|  | 1 | 65.4 | 84.5 | 37.9 | 56.6 | 95.1 |
|  | 10 | 29.7 | 56.2 | 11.4 | 15.9 | 64.0 |
|  | 25 | 18.8 | 41.8 | 9.0 | 13.5 | 49.5 |
| **EXP. 2** |  |  |  |  |  |  |
| Dose MMC | Affected | Mother | FAA |  | FAC | WT |
|  | 0 | 100 | 100 | 100 | 100 | 100 |
|  | 1 | 59.2 | 86.4 | 44.7 | 61.3 | 92.0 |
|  | 10 | 25.7 | 55.9 | 15.7 | 20.8 | 62.7 |
|  | 25 | 20.4 | 45.7 | 10.7 | 14.6 | 52.4 |
| **EXP. 3** |  |  |  |  |  |  |
| Dose MMC | Affected | Mother | FAA |  | FAC | WT |
|  | 0 | 100 | 100 | 100 | 100 | 100 |
|  | 1 | 64.9 | 73.2 | 30.6 | 44.3 | 87.5 |
|  | 10 | 35.2 | 55.9 | 18.1 | 15.6 | 61.0 |
|  | 25 | 25.3 | 42.0 | 7.1 | 9.2 | 47.7 |
| **EXP. 4** |  |  |  |  |  |  |
| Dose MMC | Affected | Mother | FAA |  | FAC | WT |
|  | 0 | 100 | 100 | 100 | 100 | 100 |
|  | 1 | 49.3 | 77.3 | 36.6 | 38.5 | 94.6 |
|  | 10 | 30.5 | 58.0 | 16.1 | 14.5 | 67.4 |
|  | 25 | 18.1 | 44.7 | 7.7 | 12.2 | 51.0 |
| **Exp. 5** |  |  |  |  |  |  |
| Dose MMC | Affected | Mother | FAA |  | FAC | WT |
|  | 0 | 100 | 100 |  | 100 | 100 |
|  | 1 | 66.7 | 86.8 |  | 63.6 | 76.7 |
|  | 10 | 38.1 | 69.1 |  | 27.3 | 60.0 |
|  | 25 | 20.2 | 55.9 |  | 15.9 | 57.0 |
| **Exp. 6** |  |  |  |  |  |  |
| Dose MMC | Affected | Mother | FAA |  | FAC | WT |
|  | 0 | 100 | 100 |  | 100 | 100 |
|  | 1 | 65.2 | 87.5 |  | 61.5 | 90.0 |
|  | 10 | 26.1 | 72.5 |  | 30.8 | 74.0 |
|  | 25 | 21.7 | 45.0 |  | 15.4 | 48.0 |
| **Exp. 7** |  |  |  |  |  |  |
| Dose MMC | Affected | Mother | FAA |  | FAC | WT |
|  | 0 | 100 | 100 |  | 100 | 100 |
|  | 1 | 64.2 | 88.9 |  | 48.3 | 85.3 |
|  | 10 | 35.8 | 60.0 |  | 21.7 | 58.2 |
|  | 25 | 25.3 | 46.7 |  | 14.2 | 45.9 |

| **MEAN GROWTH** | 0 | 1 | 10 | 25 |
| --- | --- | --- | --- | --- |
|  |
| WT | 100 | 88.7 | 63.9 | 50.2 |
| FA-A | 100 | 37.4 | 15.3 | 8.6 |
| FA-C | 100 | 51.1 | 20.2 | 13.0 |
| Mother | 100 | 83.5 | 61.1 | 46.0 |
| Affected | 100 | 62.1 | 31.6 | 21.4 |
| Affected + FA | 100 | 70.8 | 50.2 | 36.4 |
| **SD** |  |  |  |  |
| WT | 0 | 6.4 | 5.3 | 3.7 |
| FA-A | 0 | 5.8 | 2.8 | 1.6 |
| FA)C | 0 | 9.6 | 6.5 | 2.5 |
| Mot | 0 | 5.9 | 6.9 | 4.7 |
| Aff | 0 | 6.5 | 4.9 | 2.9 |
| AF+M | 0 | 1.2 | 3.7 | 5.4 |
